# Pseudoaneurysm in the abdominal wall as a rare complication following surgery of a chronically superinfected urachal malformation — An exceptional case in congenital malformation surgery

**DOI:** 10.1016/j.ijscr.2025.110979

**Published:** 2025-01-28

**Authors:** Amelie Kugler, Oliver Rompel, Manuel Besendörfer, Sonja Diez

**Affiliations:** a Pediatric Surgery, University Hospital Erlangen, Loschgestraße 15, Erlangen, Germany; b Pediatric Radiology, University Hospital Erlangen, Loschgestraße 15, Erlangen, Germany

**Keywords:** Pseudoaneurysm, Aneurysma spurium, Umbilical malformation, Persistent urachus, Pediatric surgery

## Abstract

**Introduction:**

A pseudoaneurysm, or aneurysma spurium, occurs due to a paravasal hematoma following a vascular injury. Pseudoaneurysms are extremely rare in children and adolescents. Because of the persistent blood flow through the supplying vessel, a potential rupture can cause life-threatening bleeding. Accurate diagnosis and treatment are therefore essential.

**Presentation of case:**

We present the case of an 11-month-old female infant with a congenital, exophytically growing, secreting tumor in the umbilical area. Surgical excision revealed a persistent urachus with a urachal cyst and a malformation on the bladder wall. Postoperatively, severe wound healing disorder developed, along with an intra-abdominal abscess. A pseudoaneurysm in the abdominal wall was incidentally detected with sonography, and CT confirmed perfusion from a branch of the right iliac artery. Initial catheter-based endovascular coiling was unsuccessful, and re-laparotomy for ligation, resection of the pseudoaneurysm, and debridement of the abscess was performed. The further course was complication-free.

**Discussion:**

Pseudoaneurysms, especially post-traumatic ones, can easily be misdiagnosed as a rare differential diagnosis in children and adolescents. Early diagnosis and interdisciplinary treatment are crucial for a successful outcome.

**Conclusion:**

In this particular case, it is assumed that the pseudoaneurysm developed as a result of chronic superinfection of the atypical urachal malformation.

## Highlights

• Early sectional imaging should be considered for atypical umbilical malformations.
• Surgical excision is the treatment of choice for umbilical malformations to prevent complications.
• Open excision is preferred for pseudoaneurysms, while endovascular treatment is reserved for selected cases.

## 1 Introduction

Umbilical malformations in neonates can present with a variety of symptoms, which often require careful clinical evaluation. These malformations can range from simple umbilical hernias to more complex conditions involving congenital malformations of the bladder and the intestines. Although infected urachal cysts are rare, they represent an important cause of severe sepsis in neonates, requiring early diagnosis [1].

Pseudoaneurysms, also known as aneurysma spurium, are a rare but significant medical condition in children. In this patient group, the pathogenesis is often traumatic or iatrogenic. In a study by Lin et al., pseudoaneurysms accounted for 11.8 % of all types of complications caused by iatrogenic femoral artery catheterization in children that required surgical intervention [2]. Unlike true aneurysms, which involve all layers of the arterial wall, pseudoaneurysms result from a breach in the arterial wall, typically due to trauma, surgical intervention, or infection. This breach leads to the formation of a hematoma that communicates with the arterial lumen, posing a risk of rupture and potentially life-threatening hemorrhage [3].

Early detection and treatment are essential in pediatric patients to prevent complications. In this context, we present a case report describing a pseudoaneurysm in the abdominal wall following the surgical resection of a persistent urachus in an infant, emphasizing the need for a multidisciplinary approach for accurate diagnosis and effective treatment.

This case report was written in accordance with the 2023 SCARE guidelines [4].

## 2 Presentation of case

**Fig. 1:**
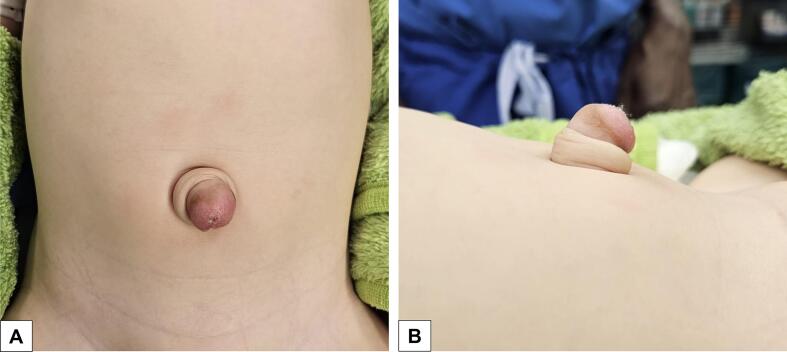
Preoperative image of the congenital, exophytically growing mass with central umbilical secretion in frontal (A) and lateral (B) view.

A 11-month-old female twin infant, prematurely born at 34 + 6 weeks of gestational age, was referred to our pediatric surgery consultation for a suspected umbilical hernia with granuloma. An exophytically growing, secreting umbilical mass with a diameter of about 2 cm had been noticed since birth [Fig. 1]. Local treatment with silver nitrate for a suspected granuloma by the pediatrician had not been effective. The patient had no history of illnesses or prior surgeries related to her prematurity. Sonographic examination revealed a sharply demarcated, subcutaneous mass with a central hypoechoic area extending beyond the abdominal fascia.

**Fig. 2:**
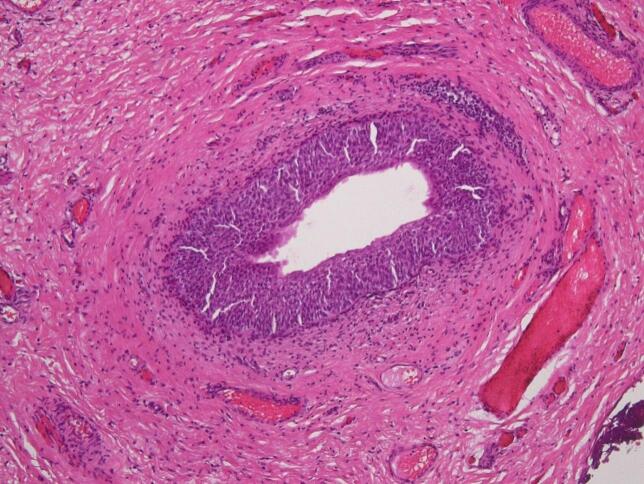
Histological slide of the excised urachal duct with urothelial epithelium.

Intraoperatively, the cyst was found in conjunction with the bladder, presenting a broad-based malformation on the upper bladder roof. The histological findings confirmed accordingly a persistent urachus with an umbilical cyst [Fig. 2]. The congenital malformation was completely removed, and the wound was primarily closed by umbilicoplasty. The patient was discharged on the second postoperative day after an initially uncomplicated course.

**Fig. 3:**
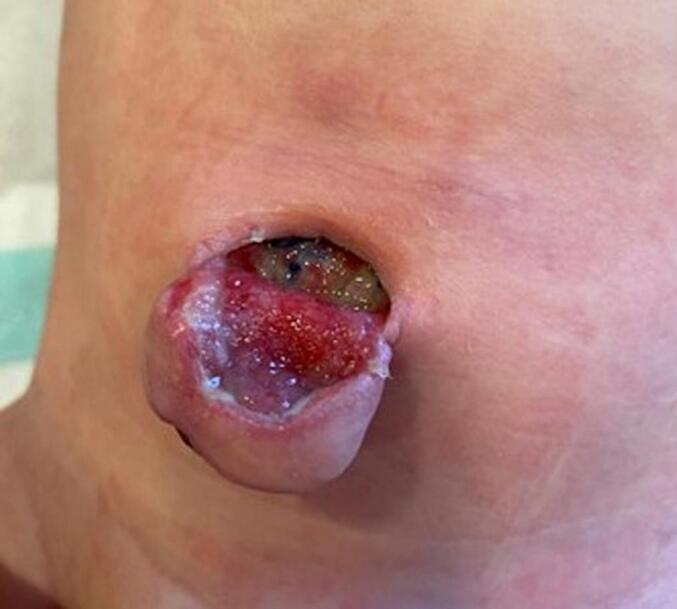
Umbilical local findings after abscess discharge.

Two days later, the patient returned to the emergency department with purulent wound secretion, extensive abdominal wall phlegmon, and fever. Accordingly, inflammatory blood values were elevated (C-reactive protein 125 mg/l, leukocytes 24,000/μl). Surgical revision revealed an umbilical abscess, which was cleaned and further treated with wound irrigation [Fig. 3]. Bacterial examination confirmed an infection with *Staphylococcus aureus*, which was sensitive to the antibacterial therapy with ampicillin/sulbactam already in progress.

Due to insufficient improvement over three days, treatment of the umbilical wound cavity was converted to continuous vacuum-assisted closure (VAC) therapy with a suction of −50 cmH_2_O. Although the local findings showed a good response, inflammatory values remained persistently elevated. The systemic treatment was intensified with piperacillin/tazobactam. Further sonographic diagnostics revealed suspicion of a supravesical intraperitoneal inflammatory process and an aneurysm spurium in the abdominal wall. From this point onward, diagnosis and treatment were therefore carried out in an interdisciplinary collaboration between pediatric surgeons, radiologists, and pediatric cardiologists.

**Fig. 4:**
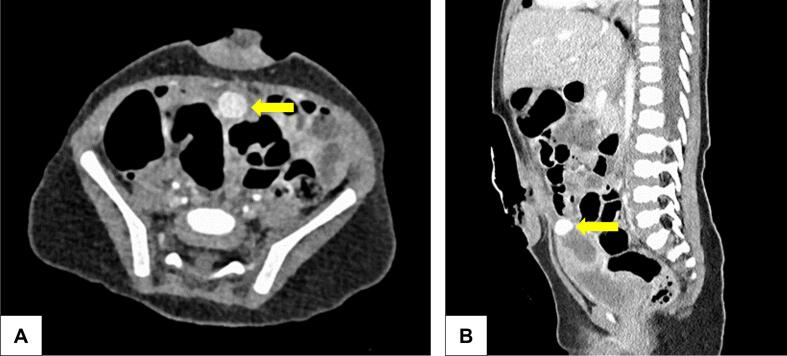
Transverse (A) and sagittal (B) CT abdomen image with contrast medium: A pseudoaneurysm with a maximum diameter of about 1 cm (yellow arrows) is visible behind the umbilical defect cavity. (For interpretation of the references to colour in this figure legend, the reader is referred to the web version of this article.)

Diagnostics were completed with an emergency computed tomography (CT) in consultation with our pediatric radiology department, confirming the findings of sonography. The pseudoaneurysm could be attributed to an abdominal wall artery feeding from the iliac artery, appearing as a strongly perfused spherical lesion with a maximum diameter of about 1 cm [Fig. 4]. Due to its size and the complex inflammatory process in the abdominal wall, it was impossible to identify the pseudoaneurysm during the superficial abscess debridement.

To minimize the risk of rupture of the pseudoaneurysm, VAC therapy was stopped. The pediatric cardiology team attempted endovascular embolization, but coiling was unsuccessful due to the small size of the feeding vessel.

An open surgical procedure was subsequently performed to ligate and resect the pseudoaneurysm, remove the surrounding hematoma, and debride the intra-abdominal abscess cavity, which was rinsed and drained for 5 days. The further postoperative course proceeded without complications. Within clinical improvement, infection values decreased under continued intravenous antibacterial therapy with piperacillin/tazobactam for further 7 days. A normal healing progression was observed, with no indications of a significant wound healing disorder. The patient was discharged on the 7th postoperative day. The further follow-up care was provided by the attending pediatrician and revealed no complications regarding the surgical aspects.

## 3 Discussion

Pediatric surgery often encounters congenital malformations that are not thoroughly discussed in standard medical textbooks. This particular case of a pseudoaneurysm in the abdominal wall, arising as a rare complication following surgery of a chronically superinfected urachal malformation, underscores the importance of comprehensive and interdisciplinary approaches in managing such atypical presentations.

Abscesses originating from urachal remnants or ductus omphaloentericus are uncommon but significant causes of severe infections in neonates and infants. It is thought that most urachal cysts are asymptomatic and are discovered incidentally during imaging performed for unrelated conditions [5,6]. However, in the rare instances where these anomalies become symptomatic, they can present variably in both children and adults. The symptoms are non-specific and range for example from painless umbilical discharge of urine or stool in infants to hematuria, periumbilical mass, abdominal pain accompanied by fever, erythema or infection in older children and adults [[5], [6], [7], [8]]. This variability often complicates prompt diagnosis and treatment. Despite the variable symptoms, most authors state that clinical findings alone are sufficient to guide an accurate diagnosis in most cases [5,9,10]. Ultrasound is considered the imaging method of choice for confirmation, with a reported sensitivity of up to 100 % for detecting urachal remnants, according to Yiee et al. [9]. If an ultrasound remains non-diagnostic and there is no umbilical discharge, advanced imaging modalities such as CT or MRI are indicated for further diagnostic evaluation [5,9,10]. As an important complication, persistent urachal cysts, like the one seen in this case, can become superinfected, leading to severe systemic infections such as sepsis, or can rupture, resulting in peritonitis [11]. Consequently, early identification and an appropriate treatment are crucial to mitigate these potentially life-threatening complications. Despite the low prevalence of this condition, the aforementioned symptoms should always prompt consideration of a urachal remnant as a potential differential diagnosis, particularly in the pediatric population. Considering the potential for malignant transformation and fistula or abscess formation of a urachal lesion, a systematic complete surgical excision after diagnosis seems advisable for a definite treatment and safe outcome [1,7,8,12].

Pseudoaneurysms, while rare in pediatric patients, are a known complication following trauma, surgical procedures, or infections in adults. Unlike true aneurysms that involve all layers of the arterial wall, pseudoaneurysms result from a breach in the arterial wall, leading to a contained hematoma that maintains communication with the arterial lumen. This condition poses a high risk of rupture and subsequent hemorrhage [13]. In adults, pseudoaneurysms are more frequently reported and better understood [[14], [15], [16]]. The most common cause is the iatrogenic pseudoaneurysms of the femoral artery following endovascular interventions [17]. According to Henry et al., the incidence is estimated to range from 2.9 % to 3.8 % among patients who undergo catheterization via femoral access [15]. However, in children, the rarity of such cases often leads to delayed recognition and treatment, emphasizing the need for heightened clinical vigilance for early imaging modalities [18]. The existing literature focuses primarily on cases of pseudoaneurysms in children that occur following arterial punctures [19], endovascular interventions [2,20], or penetrating trauma [13,18], typically localized to the extremity or to the cervical arteries. In cases of clinical suspicion of a pseudoaneurysm, characterized by a pulsatile mass in the extremities, Doppler ultrasound should be promptly performed as the initial imaging modality. To obtain a more detailed view of the feeding vessel and surrounding structures, further cross-sectional imaging with CT or MRI angiography is strongly recommended [15,20]. Overall, timely and comprehensive imaging is essential for accurate diagnosis and optimal treatment planning of pseudoaneurysms. Depending on the size and location, different therapeutic modalities are available. In the reported cases concerning children, open surgical excision of the hematoma followed by vascular suturing is described as the definitive treatment [13,20]. According to Henry et al., endovascular interventions are considered a suitable therapeutic option only in very selected cases [15]. In this instance, the interdisciplinary decision-making process led to the choice of endovascular coiling due to its lower invasiveness and reduced risk compared to open surgical treatment. Even if this attempt was unfortunately unsuccessful due to the extremely small size of the feeding vessel, this proves the importance of an interdisciplinary team of specialists (pediatric surgery, pediatric radiology, pediatric cardiology) to provide optimal therapeutic options in individual cases.

To the best of our knowledge, we present the first published case of a postoperative pseudoaneurysm in the abdominal wall arising after surgery of a chronically infected urachal malformation. We acknowledge the fact, that this case report is unable to present evidence-based recommendations in the treatment of urachus malformations. However, this case was associated with several clinical challenges, which might help to solve further similar cases. The atypical presentation of the congenital malformation with severe wound healing disorders suggested the need for early advanced imaging modalities. Magnetic Resonance Imaging (MRI) could have provided earlier and more detailed insights into the extent of the underlying pathology. We estimate the decision against it now as a limitation of this case. However, the decision to perform MRI must be balanced against the risks associated with anesthesia in infants. In this case, Computed Tomography (CT) emerged as a viable and readily available alternative, providing detailed imaging with low-dose protocols specifically tailored for pediatric patients.

The successful management of this case underscores the importance of interdisciplinary collaboration. The close cooperation between pediatric surgeons, radiologists, and pediatric cardiologists facilitated a comprehensive evaluation and effective treatment strategy. The advantage of a university hospital setting, with access to a wide range of specialties and advanced diagnostic tools, cannot be overstated. This multidisciplinary approach ultimately resulted in a positive outcome for the patient.

Pediatric surgeons must remain prepared to encounter rare and complex cases. Anticipating unusual complications, such as pseudoaneurysms following surgery for congenital malformations, is essential. This case emphasizes the importance of continuous education and vigilance in recognizing atypical presentations and maintaining a high index of suspicion for rare but potentially severe complications.

## 4 Conclusion

In conclusion, this exceptional case of a pseudoaneurysm arising from a chronically superinfected urachal malformation underscores the importance of early diagnosis, interdisciplinary collaboration, and the appropriate use of advanced imaging modalities. Pediatric surgeons should remain vigilant and prepared for atypical presentations, leveraging the strengths of a multidisciplinary team to ensure optimal patient outcomes.

## Consent for publication

Written informed consent was obtained from the patient's parents for publication of this case report and accompanying images. This report does not contain any personal information that could lead to the identification of the patient.

## Ethical approval

The study is exempt from ethical approval in our institution.

## Funding

This research did not receive any specific grant from funding agencies in the public, commercial, or not-for-profit sectors.

## Author contribution

Amelie Kugler: corresponding author, collection of data, conception and revision of the

manuscript.

Oliver Rompel: data collection, review of the manuscript.

Manuel Besendörfer: data interpretation, guarantor, review of the manuscript.

Sonja Diez: acquisition of data, conception and revision of the manuscript

## Guarantor

Prof. Dr. med. Manuel Besendörfer.

## Research registration number

Our manuscript is a case report not a research.

## Declaration of competing interest

None to declare.
